# Supplementary material for: Time Makes Space: Emergence of Place Fields in Networks Encoding Temporally Continuous Sensory Experiences
Source: ArXiv. 2025 Jan 29:arXiv:2408.05798v2. Preprint. [Version 2] (PMC11838703)
Supplement: Supplementary file 1 [file NIHPP2408.05798v2-supplement-1.pdf]

Supplemental Materials

1 Training details

1.1 Simulated trials and the artificial agent

An artificial agent explores simulated room(s) defined by fixed WSM signals, as defined in Section 2.2. The agent updates its behavior and samples sensory experience every 50ms. To realistically model rodent movement trajectories, we simulate a random walk, where artificial agents explore the room in a trajectory with random small drifts representing an animal's exploration interest: at every timestep, the agent randomly changes its movement speed and direction with a small probability, 0.2 and 0.3, respectively. The change in speed is randomly chosen from an $\mathcal{N}(5, 1)$ cm/s normal distribution, and the drift direction is randomly chosen from $\mathcal{N}(0, 0.05)$ rad/s.

1.2 Training Recurrent Autoencoder (RAE)

The simulated artificial agent gathers experience vectors along its trajectory. We train the RAE on 1s experience segments with a batch size of 500, sampled from a 300s 'decaying' distribution of recent events to encourage the network update in consideration of an extended history of events. For an event that is t seconds away from the current time step, the probability of it being sampled is:

$$P(t) = \frac{\left(\frac{T_s - t}{T_s}\right)^{\alpha} + \beta}{\sum_{j=1}^{T_s} \left(\frac{j}{T_s}\right)^{\alpha} + \beta} \tag{1}$$

Where T_s is the width of the sampling window, $\alpha = 3$ and $\beta = 0.5$.

Our RAE contains three layers: The first and the third layers represent the signals being projected to and read out from CA3, respectively. The hidden layer, being fully connected by recurrent connections, corresponds to CA3. The hidden-layer states, which model activation potentials of neurons, are governed by the following continuous-time equation:

$$\tau \frac{d\mathbf{v}_t}{dt} = -\mathbf{v}_t + \mathbf{W}^{rc} f(\mathbf{v}_t) + \mathbf{W}^{in}\mathbf{e}'_t + \mathbf{b} + \boldsymbol{\eta}_t \tag{2}$$

Here $f(x)$ is an activation function and τ governs the decay. $\mathbf{W}^{in}$ is the connection matrix to the hidden layer, simulating pathways entering the hippocampus. $\mathbf{W}^{rc}$ is the recurrent connectivity matrix emulating CA3 recurrent collaterals. The vector $\mathbf{b}$ is a firing bias term and $\boldsymbol{\eta}_t$ denotes Gaussian pre-activation noise. The variable $\mathbf{v}_t$ models neural activation potentials. Setting $\gamma = \frac{dt}{\tau}$ and adapting to discrete- time gives:

$$\mathbf{v}_{t+1} = \mathbf{v}_t + \Delta\mathbf{v}_t = (1-\gamma)\mathbf{v}_t + \gamma\left[\mathbf{W}^{rc}\mathbf{h}_t + \mathbf{W}^{in}\mathbf{e}'_{t+1} + \mathbf{b} + \boldsymbol{\eta}_{t+1}\right] \tag{3}$$

$$\mathbf{h}_t = f\left(\mathbf{v}_t\right) + \boldsymbol{\xi}_t \tag{4}$$

Here we have replaced $f\left(\mathbf{v}_t\right)$ in equation (2) with equation (4), where $\mathbf{h}_t$ is the state vector of the hidden layer neurons in Hertz (Hz) and $\boldsymbol{\xi}_t$ is the post-activation noise.

The network is trained with mean-squared error without strict sparseness constraints. The loss function is:

$$\mathcal{L} = \frac{\lambda_{mse}}{D \cdot T \cdot B} \sum_{d,t,b}^{D,T,B} \left(\hat{e}_{d,t,b} - e_{d,t,b}\right)^2 + \frac{\lambda_{fr}}{N} \sum_{n}^{N} \left(\sum_{t,b}^{T,B} r_{n,t,b}\right)^2 \tag{5}$$

D is the dimension of the Experience Vector (EV), T measures timesteps within a memory segment, B indicates the number of batches, and N counts nodes in the hidden layer. The term $\hat{e}_{d,t,b}$ is the d-th entry of the reconstructed EV at timestep t in batch b, while $e_{d,t,b}$ is the equivalent entry of the ground truth. The second component of the loss, $r_{n,t,b}$, denotes the firing rate of the n-th neuron at time t within batch b. Unless otherwise stated, we set $\lambda_{mse} = 0$ and $\lambda_{fr} = 200$ for our experiments.

Table 1: Network parameters

Parameter	Description	Value
n_hidden	# hidden layer nodes	1000
InputLayer	Distribution of the input layer	$\mathcal{U}(-\sqrt{k}, \sqrt{k})$, where $k = \frac{1}{\text{in\_features}}$
HiddenLayer	Distribution of the hidden layer	$\mathcal{U}(-\sqrt{k}, \sqrt{k})$, where $k = \frac{1}{\text{n\_hidden}}$
OutputLayer	Distribution of the output layer	$\mathcal{U}(-\sqrt{k}, \sqrt{k})$, where $k = \frac{1}{\text{n\_hidden}}$
dt	Time resolution	50 ms
τ	Time constant	500
$\gamma = \frac{dt}{\tau}$	Decaying factor	0.1
Optimizer	Network optimizer	Adam
learning_rate	The learning rate of RAE	0.0005
λ_{mse}	Coefficient for the mean-squared pattern completion error	1
λ_{fr}	Coefficient for the mean-squared hidden layer firing rates	200

Table 2: Training parameters

Parameter	Description	Value
N_{batch}	Number of episodic memory in each 'bout'	500
warmup_duration	The duration of experience vector before the first trial room (see Fig. 1b)	300 s
masking_ratio	The percentage we use to randomly occlude an arbitrary experience vector	$\mathcal{U}(r_{min}, r_{max})$ $r_{min} = 0$ & $r_{max} = 0.2$
α	The exponential coefficient in equation (1)	3
β	The constant term in equation (1)	0.05
spatial_resolution	How each meter in the real-world is converted into pixels in our simulated rooms	1 cm/pixel
T_w	Width of the sampling window (see Fig. 1 caption)	300 s
T_s	Length of each episodic memory	1 s
Δt	The step size of how much to move the sampling window forward	1 s

1.3 Testing RAE and visualizing hidden layer firing profiles

After training in each trial, we paused parameter updates and “recorded" the network response at every location in the room. The network continued to receive batches of partially occluded experiences with added noise, generated by a random traveling agent, as detailed in the main text. To ensure sufficient data at every location and to average out effects of random occlusions and noise, we conducted 20-minute trials repeated 20 times. Importantly, the obtained spatial maps are robust to both the duration and the number of tests since we paused parameter updates during testing.

During these tests, we recorded the firing rate (post-activation value) of each hidden layer unit at every location. We then averaged the unit's responses at each location across all previous visits. We repeated this process for all locations and hidden layer units to obtain spatial maps after.

2 Measurements and metrics

2.1 Spatial information content

We used the Spatial Information Content (SIC) [51] measured in bits to measure the strength of a cell's place-like property in terms of its spatial selectivity We discretize firing rate maps into M 30cm $\times$ 30cm bins. Each bin has a firing rate and associated probability p_m of the cell firing. The spatial

information content (SIC) is computed as:

$$SIC = \sum_{m=1}^{M} p_m \cdot (\frac{r_m}{\bar{r}}) \cdot log_2(\frac{r_m}{\bar{r}})$$

where $\bar{r}$ is the average firing rate across bins. Bins with $r_m = 0$ are skipped in the sum (to avoid divergences in the log), but are included in the computation of the average rate $\bar{r}$.

2.2 Synapse reorganization score

We quantify synaptic adaptation as the ratio of the Frobenius norm of the change in the weight matrix to the Frobenius norm of the weight matrix prior to update:

$$\frac{||W^{old} - W^{new}||_F}{||W^{old}||_F}$$

Here, W^{old} and W^{new} represent synaptic weight matrices before and after adaptation, respectively.

3 Orthogonal spatial representations

3.1 Measurement of orthogonality

To measure how generated place-like patterns form representations of different rooms, and to what extent these representations are orthogonal, we adopted a methodology similar to that proposed by Alme et al. [21]. At the end of each trial, we converted the 100 $\times$ 100 cm firing rate map from a single unit into a 20 $\times$ 20 grid by averaging the rates within each bin to obtain a population coding vector for a room. To compute the similarity between two population vectors, X and Y, Alme et al. [21] used the mean dot product:

$$\frac{1}{N \cdot W \cdot H} \sum_{i,j,k}^{N,W,H} X_{i,j,k} Y_{i,j,k}$$

Here N is the number of units, and W and H are the bin dimensions in width and height, respectively. The dot product involves the population vector of a room as a whole. However, to provide a normalized measure of similarity for Fig. 5 in our main paper, we adopted the Pearson correlation. Pearson correlation also uses the population vector of a room as a whole, but normalizes the data to reduce the dominance of highly active units. Thus, the similarity between X and Y is calculated as:

$$\frac{\sum_{i=1}^{N \cdot W \cdot H}(X_i - \overline{X})(Y_i - \overline{Y})}{\sqrt{\sum_{i=1}^{N \cdot W \cdot H}(X_i - \overline{X})^2}\sqrt{\sum_{i=1}^{N \cdot W \cdot H}(Y_i - \overline{Y})^2}}$$

3.2 Drifting place fields and orthogonal representations

To test whether the generated representations can be used to construct cognitive maps for different enclosures, and whether these maps can be maintained over an extended period, we placed the agent in 20 rooms for 30 cycles, resulting in 600 recordings. In the main text, we compared how spatial representations in the last cycle compared to representations from previous cycles. Here, we offer a more comprehensive comparison. Suppl. Fig. 1 presents the cross-comparison of all 600 recordings, showing that recordings from the same room remain correlated even many cycles apart. Only recordings from cycle 1 show a slightly decreased correlation score, due to the incomplete formation of place fields.

As the simulated agent travels through multiple rooms over an increasing number of cycles, we observe that place cells, while reverting to their previous locations upon reentering a familiar room, may exhibit slight shifts in their locations. As the number of cycles between two trials of a single room increases, this discrepancy becomes increasingly pronounced, manifesting as a place field drift. Figure 2 depicts the drift of six randomly selected hidden layer units across 30 cycles in Room 1.

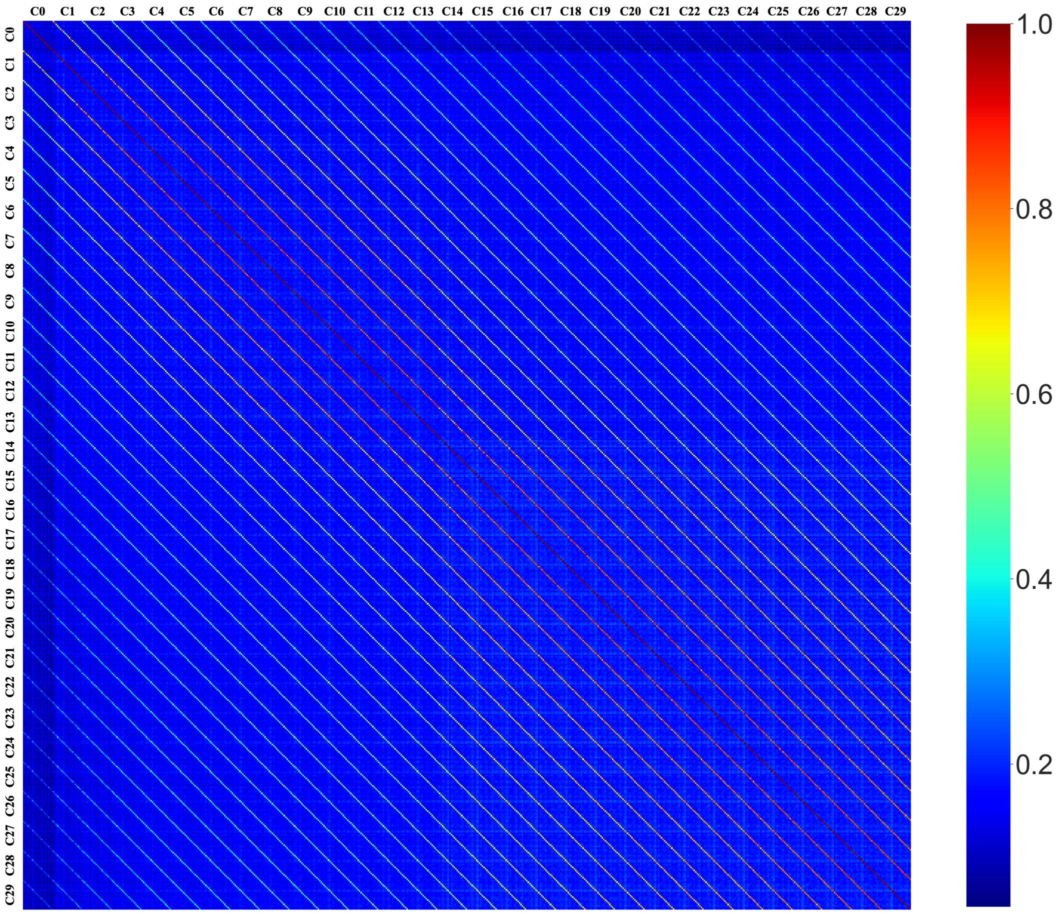

Figure 1: Cross correlation of all 600 recorded trials. Each pixel represents a comparison of two trials. During each cycle, the sequence in which the agent explores the 20 rooms is shuffled. We re-organize the room sequence when plotting the cross-correlation between trials to ease visualization. The periodic lines indicate a strong correlation of spatial representations generated when the animals entered the same room, even in different cycles.

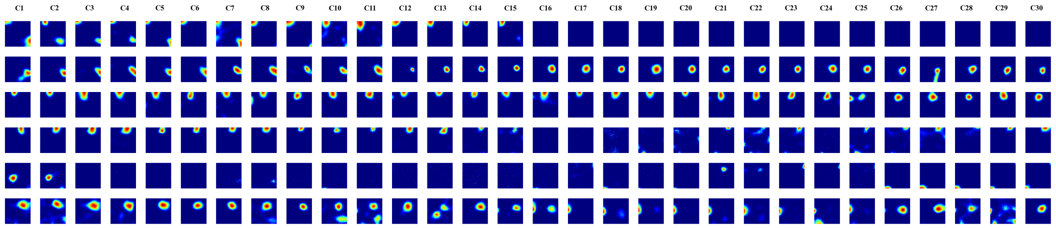

Figure 2: Illustration of place field drift across multiple visits to the same room over 30 cycles.

4 Additional results

4.1 Pattern completion results of RAE

We train the RAE to reconstruct the complete, noiseless experiences from partial and noisy sensory inputs. To examine pattern-completion performance, we pause training after each trial to record the reconstructed values and compute the average firing rate at each location for each output channel. This process is similar to the method used to gather firing profiles from the hidden layer. In Suppl. Fig. 3,

we plot 20 randomly selected WSM cells and their reconstructed values. All outputs accurately reflect the sensory changes appearing in the original WSM signals.

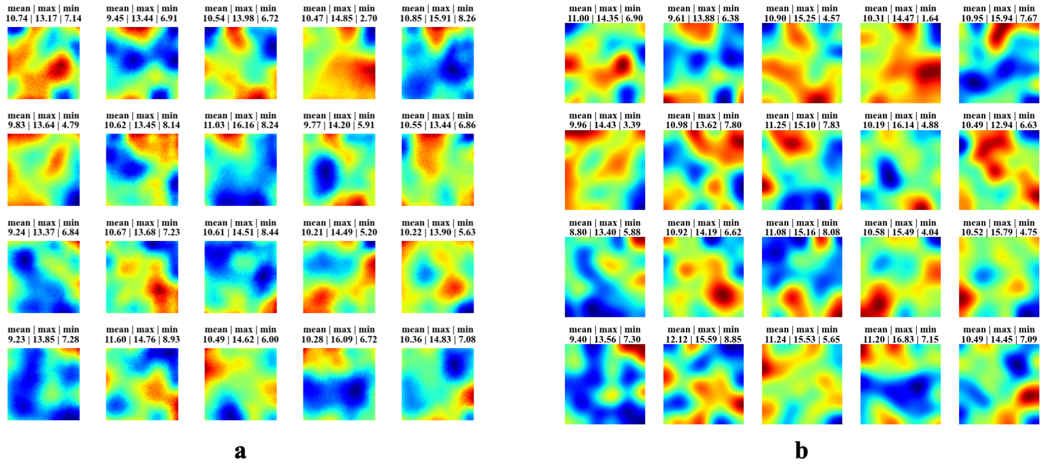

Figure 3: Example pattern completed WSM signals and their corresponding ground truths. **a.** 20 randomly selected WSM signals reconstructed by the RAE and plotted on the 2D space map. **b.** The ground truth values corresponding to the WSM cells in panel a.

4.2 Robustness to parameter variations

We have verified that the emergence of place cells is consistent across different environment shapes, for example triangles (Suppl. Fig. 4a) and circles (Suppl. Fig. 4b). In both cases, the hidden layer generated place-like patterns similar to those in the square rooms we used in our experiment.

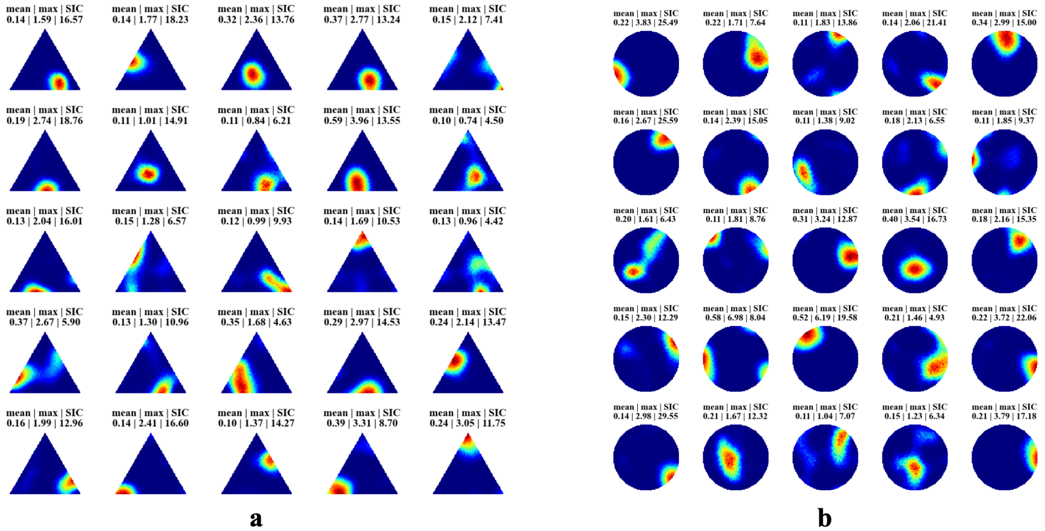

Figure 4: **a.** Example of emerged place-like patterns in a triangular room. **b.** Example of emerged place-like patterns in a circular room.

While we fixed the parameter values during our experiments to enhance reproducibility, we also verified that place cell emergence is robust to parameter changes and does not require specific values. To quantify, we evaluated the firing maps of hidden layer units using three metrics: (1) the percentage of active units, defined as units with a maximum firing rate > 0.1 Hz; (2) the percentage of place units, identified by a Spatial Information Content (SIC) > 5; (3) the average SIC across all active units.

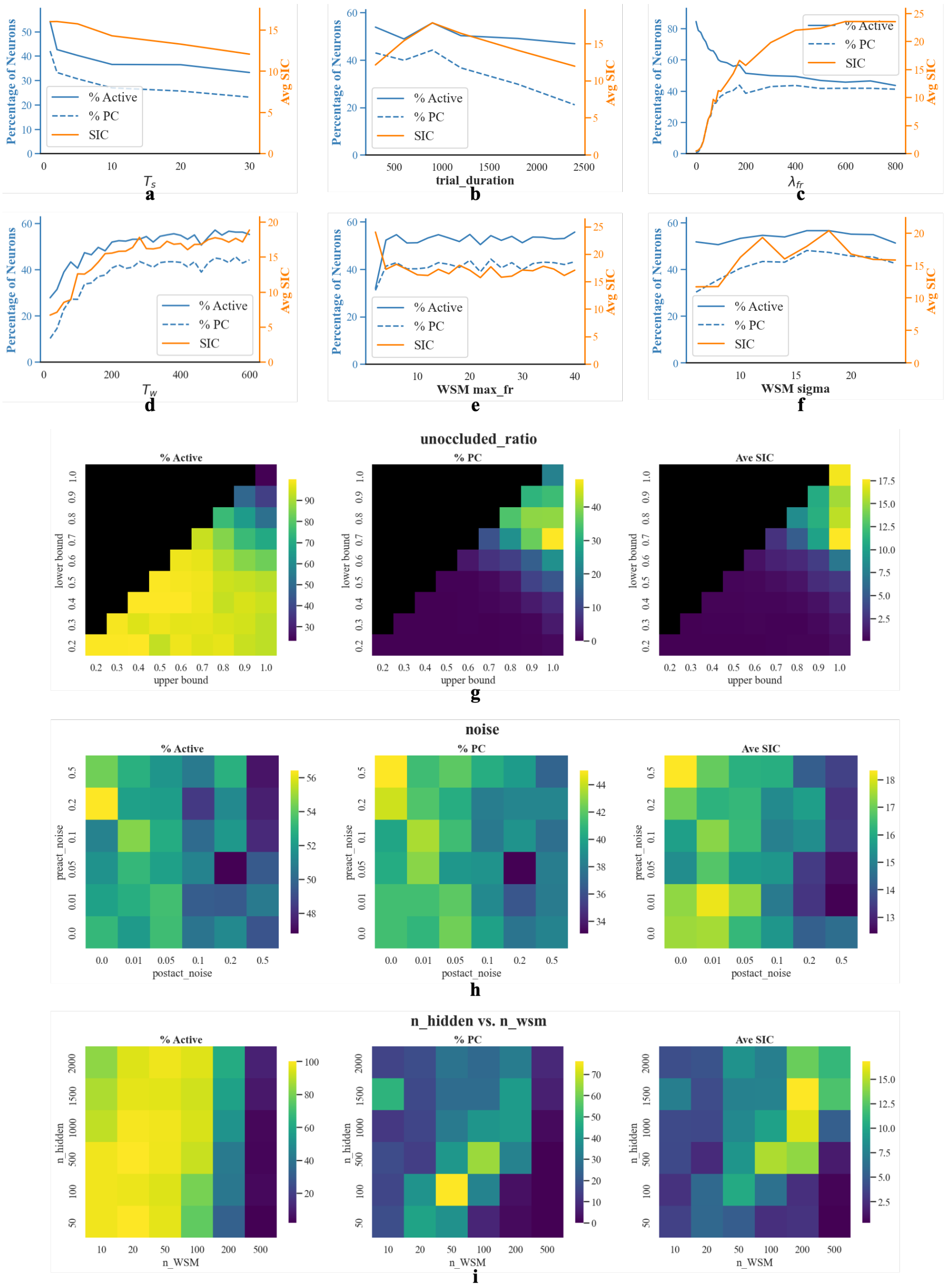

Figure 5: **a-i.** Impact of varying specific parameters, with others held constant at the default values, on place cell emergence. All experiments are conducted in a single trial room. Evaluation of hidden layer firing rate maps included three metrics: percentage of active cells ($max\_fr > 0.1$ Hz) among all hidden units, percentage of place cells ($max\_fr > 0.1$ Hz and SIC > 5) among all hidden units, and average SIC across active units. **a.** Duration of episodic memory segments. **b.** Total trial duration. **c.** Coefficient of firing rate loss. **d.** Warmup trial length (the recall length is also set to the same value). **e.** Maximum firing rate for each WSM channel. **f.** Sigma value for smoothing WSM signals. **g.** Ratio of unoccluded experience. We randomly preserve a fraction of the experience and train the RAE to recall the masked part, where the fraction is drawn uniformly in the interval $[r_{min}, r_{max}]$. Here we vary the bounds on this interval. **h.** Effects of pre-activation and post-activation noise. **i.** Co-varying the number of hidden units and the number of WSM cells (dimension of EV).

Figure 5 shows that: (1) Increasing the duration of each episodic memory segment slightly reduces the number of place cells, as longer episodes likely involve multiple locations, decreasing spatial specificity. Despite this, the majority of active cells continue to exhibit place-like characteristics. (see Fig. 5a). (2) The number of place cells decreases slowly as trial duration increases. This decrease is due to the optimizer forcing the network to encode WSMs more efficiently after the MSE loss stops decreasing. This optimized encoding is thus overfitted to one single room and requires individual cells to fire at unrealistic rates, which are unlikely to occur in biological systems (see Fig. 5b) (3) As λ_{fr} increases, all active cells become place cells. (4) The number of place cells and active cells increases as the recall length increases, stabilizing once the recall duration exceeds 200 seconds. (see Fig. 5d) (5) Neither the maximum firing rate of WSM signals nor the sigma value affects the emergence of PFs. (see Fig. 5e & 5f) (6) Place fields only emerge when the number of WSM signals exceeds 100, aligning with our hypothesis in the main text that the emergence of place fields requires a large number of WSM signals. We observed in our experiments that the number of hidden units required for PF emergence increases as the dimension of the experience vector (EV), i.e., the number of WSM signals, increases. This is likely because increasing the dimensionality of experience will increase the amount of information that must to be stored, and thereby require more hidden units. We leave exploration of the best ratio between input and hidden units for future work. Overall, our main finding is that the emergence of place fields is robust under a wide range of parameters.

4.3 Attractor landscape of the RAE model of place cells

Classical theories of CA3 episodic memory storage suggest that the recurrent connections establish attractor dynamics [29, 30, 28, 68]. Each attractor basin can be either a singular fixed point or a collection of points in the network's state space. Nearby network states evolve autonomously toward these attractors [47]. The attractor network theory of episodic memory also accounts for spatial memory storage, where each place-cell firing field corresponds to an attractor basin. Therefore, we also examined the attractor landscape of our RAE.

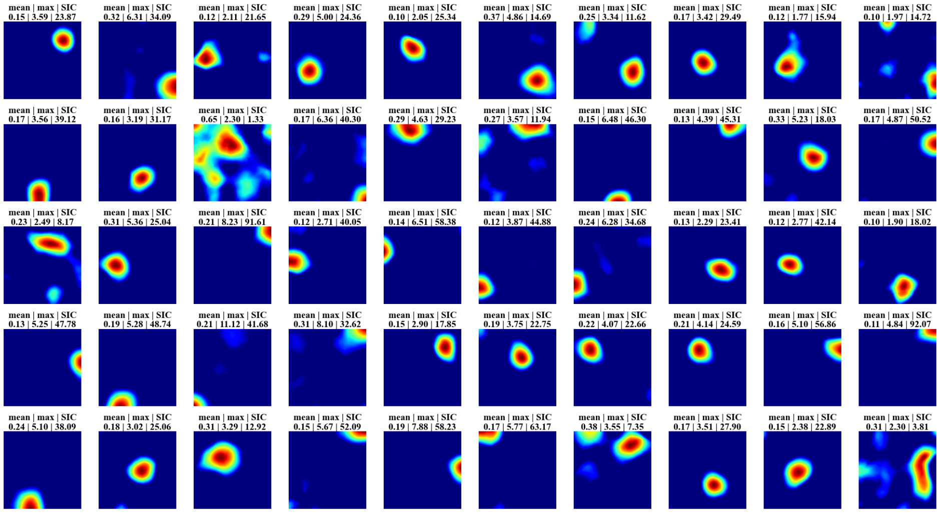

Figure 6: Example of neuronal responses at convergence points. For each location, we sample its specific EV, pause synaptic updates, and test the network by fixing this EV until convergence. Neuron states at each location are recorded upon convergence.

Attractor basins are locations where the network state becomes stable or dynamically stable. We thus examined how the network evolves when presented with a constant input. For this analysis, we simplified the network update function by removing the noise and bias terms:

$$\mathbf{v}_{t+1} = \Phi(\mathbf{v}_t) = (1-\alpha)\mathbf{v}_t + \alpha\left[W^{rc} f(\mathbf{v}_t) + W^{in}\mathbf{e}^*\right] \tag{6}$$

where we removed the bias and noise terms to simplify the calculation. As we show below, the network state update function is a contraction, and if inputs are held constant, the network will converge to a stable state. Given this property, we can plot the attractor landscape of our RAE

network by testing the network with location-specific EVs, and waiting for convergence to the attractor at each point.

As depicted in Suppl. Fig. 6, individual units maintain place-like patterns after the network has converged. This single-unit level continuous response at convergence will also result in a continuous landscape after the network converges. Thus, our network also aligns with the classical attractor theory of place cells.

Proof. If the input to the RNN is fixed (i.e., $\forall i, j \in [0, T], i \neq j$, we have $\mathbf{e}_i = \mathbf{e}_j = \mathbf{e}^*$), where T is the length of the input sequence. We would like to prove the network update function is a contraction, i.e., the network will converge to a fixed state. We work with the network update function:

$$\mathbf{v}_{t+1} = \Phi(\mathbf{v}_t) = (1-\alpha)\mathbf{v}_t + \alpha \left[W^{rc} f(\mathbf{v}_t) + W^{in}\mathbf{e}^*\right] \ . \tag{7}$$

To show that the network will converge to a fixed state, we first show that the network update function is a contraction. To do so, we need to show $\forall i, j \in [0, T], i \neq j$, if $\|\Phi(\mathbf{v}_j) - \Phi(\mathbf{v}_i)\| < k\|\mathbf{v}_j - \mathbf{v}_i\|$, where $k < 1$.

Suppose $\mathbf{v}_j - \mathbf{v}_i = \mathbf{u}$, then

$$\Phi(\mathbf{v}_j) = \Phi(\mathbf{v}_i + \mathbf{u}) = (1-\alpha)(\mathbf{v}_i + \mathbf{u}) + \alpha \left[W^{rc} f(\mathbf{v}_i + \mathbf{u}) + W^{in}\mathbf{e}^*\right] \tag{8}$$

$$\begin{aligned} \Phi(\mathbf{v}_j) - \Phi(\mathbf{v}_i) &= (1-\alpha)\mathbf{v}_j + \alpha \left[W^{rc} f(\mathbf{v}_j) + W^{in}\mathbf{e}^*\right] - (1-\alpha)\mathbf{v}_i - \alpha \left[W^{rc} f(\mathbf{v}_i) + W^{in}\mathbf{e}^*\right] \\ &= (1-\alpha)\mathbf{u} + \alpha W^{rc}\left[f(\mathbf{v}_i + \mathbf{u}) - f(\mathbf{v}_i)\right] \end{aligned} \tag{9}$$

By the triangle inequality and submultiplicativity,

$$\|\Phi(\mathbf{v}_j) - \Phi(\mathbf{v}_i)\| \leq (1-\alpha)\|\mathbf{u}\| + \alpha\|W^{rc}\|\|f(\mathbf{v}_i + \mathbf{u}) - f(\mathbf{v}_i)\| \tag{10}$$

Furthermore, if the activation function is bounded and is Lipschitz continuous on real numbers, a Lipschitz constant L exists such that $\|f(\mathbf{v}_i + \mathbf{u}) - f(\mathbf{v}_i)\|$ is bounded by $L\|\mathbf{u}\|$. Thus,

$$\begin{aligned} \|\Phi(\mathbf{v}_j) - \Phi(\mathbf{v}_i)\| &\leq (1-\alpha)\|\mathbf{u}\| + \alpha\|W^{rc}\|L\|\mathbf{u}\| \\ &= (1-\alpha+\alpha\|W^{rc}\|L)\|\mathbf{u}\| = k\|\mathbf{u}\| \end{aligned} \tag{11}$$

The update function is a contraction mapping if $k = (1-\alpha) + \alpha\|W^{rc}\|L < 1$. Therefore, $\|W^{rc}\| < L^{-1}$ to ensure $k < 1$. The Lipschitz constant is $L = 0.25$ for sigmoid activation, $L = 0.5$ for tanh activation, and $L = 1$ for the positive part of ReLU activation.

Because the neuronal activation potentials are real numbers and we are using the distance function as the metric, the state-space is complete. By the Banach fixed-point theorem, iteratively applying this transformation will converge to a fixed point $\mathbf{v}^*$ in the state space. □
